# INCIDENCE AND DYNAMICS OF MOBILITY DEVICE USE AMONG COMMUNITY-DWELLING OLDER ADULTS IN THE UNITED STATES

**DOI:** 10.1093/geroni/igad104.3428

**Published:** 2023-12-21

**Authors:** Xinran Liu, Sara Baumann, Andrea Rosso, Elizabeth M Venditti, Yao Yao, Steven Albert

**Affiliations:** University of Pittsburgh, Pittsburgh, Pennsylvania, United States; University of Pittsburgh, Pittsburgh, Pennsylvania, United States; University of Pittsburgh, Pittsburgh, Pennsylvania, United States; University of Pittsburgh, Pittsburgh, Pennsylvania, United States; Peking University Health Science Center, Beijing, Beijing, China (People’s Republic); University of Pittsburgh, Pittsburgh, Pennsylvania, United States

## Abstract

Mobility devices significantly enhance the activities and participation for individuals with mobility disability, particularly in the rapidly expanding population of older adults globally. This research reports patterns of mobility device use among community-dwelling older adults in the United States. Using the National Health and Aging Trends Study (NHATS) waves 1-9, data on basic demographics and mobility device use were collected in a nationally representative group of community-dwelling older adults (65+ years old). Mobility device use included canes, walkers, wheelchairs, and scooters. In a total of 47,722 person-years observed in community settings, 2,943 incident mobility device uses over an average of 3.8±3.0 years were identified among 2,591 participants, with an incidence rate of 61.7/1,000 person-year. Participants had an average age of 80.3±7.2 years, with 57.7% female, 70.2% White, 20.1% Black, and 5.7% Hispanic, and 34.6% living alone. The majority (51.3%) of incident mobility device use lasted no longer than one year, and only 18.6% continued for four or more years. The most popular combinations of mobility device types were cane only (44.3%), cane+walker (15.7%), walker only (15.1%), and walker+wheelchair (8.3%). 30.5% of community-dwelling older adults changed their patterns of mobility device use at least once, with the most common changes including cane only to cane+walker (16.4%), cane+walker to cane only (7.97%), walker only to walker+wheelchair (5.2%), and cane+walker to walker only (5.1%). Mobility device use among community-dwelling older adults is dynamic: use of mobility devices involves mostly short bouts and changes frequently, with a preference for a combination of mobility devices.

